# Molecular mechanisms driving lineage plasticity in prostate cancer: NANOG and beyond

**DOI:** 10.47248/chp2502010001

**Published:** 2024-12-27

**Authors:** Hamed Maasoumyhaghighi, Mansoureh Nouri, Jinghui Liu, Xiaoqi Liu

**Affiliations:** 1. Department of Toxicology and Cancer Biology, University of Kentucky, Lexington, KY 40536, USA; 2. Markey Cancer Center, University of Kentucky, Lexington, KY 40536, USA

**Keywords:** NANOG, lineage plasticity, stemness, castration-resistant prostate cancer (CRPC), neuroendocrine prostate cancer (NEPC), androgen receptor (AR), cancer stem cells (CSCs), polo-like kinase 1

## Abstract

Developing resistance to androgen receptor (AR) signaling inhibitors is a significant challenge in the treatment of castration-resistant prostate cancer. Prolonged use of inhibitors like enzalutamide can cause prostate cancer cells to undergo lineage reprogramming, transitioning to neuroendocrine subtypes that no longer rely on AR signaling. These neuroendocrine subtypes are among the most aggressive forms of prostate cancer. During this process of lineage plasticity, cancer cells experience extensive transcriptional rewiring and acquire stem-like properties characterized by increased stemness. Research has shown that prostate cancer cells gain these stem-like traits through the expression of stem cell-associated proteins such as NANOG, particularly under stable and accumulating conditions. The post-translational modification of NANOG at specific sites is critical for maintaining its stability, which in turn enhances the tumorigenic potential of the cells. This review discusses the mechanisms by which NANOG phosphorylation promotes stemness and lineage plasticity in prostate cancer.

## 1. Introduction

Prostate cancer is projected to account for 29% of newly diagnosed cancer cases among American males in 2024, according to the latest cancer statistics. It is the second leading cause of cancer-related mortality in men, following lung cancer [1]. Androgen steroid hormones in prostate cancer attach to the androgen receptor (AR), initiating a crucial carcinogenic transcriptional pathway specific to a certain lineage. This fact has been used therapeutically for many years to treat recurring metastatic disease following local therapy like initial surgery or radiation therapy. While first-generation competitive AR inhibitors or androgen deprivation therapy temporarily stop tumor growth, most patients eventually become resistant to the treatment, which leads to the development of castration-resistant prostate cancer (CRPC) [2,3]. Enzalutamide (ENZA) is a second-generation AR blocker that has demonstrated increased survival rates and has been licensed for the treatment of aggressive CRPC. Nevertheless, most patients develop resistance to enzalutamide due to gene amplification and gain-of-function point mutations on the AR gene, which reactivates AR signaling [3,4]. Meanwhile, some tumors can reprogram to a different lineage while no longer relying on AR signaling, for instance, neuroendocrine, also referred to as small cell PCa, which belongs to the most aggressive kinds of prostate cancer. Lineage plasticity allows cancer cells to change their biological phenotype and is associated with extensive rewiring of transcription, frequently linked to more advanced stages of the disease. This lineage shift allows cancer cells to escape AR pathway inhibitors. The growing usage of powerful ARPIs has led to an increase in treatment-induced NEPC. This group of patients makes up 20% of advanced, treatment-resistant CRPC.

Embryonic stem cells (ESCs) and cancer stem cells share several characteristics, including the expression of pluripotency-related transcription factors such as NANOG, OCT4, and SOX2. High expression levels of these transcription factors in cancer cells are strongly correlated with the initiation and progression of various cancer types [5]. The transcription factor NANOG, which is associated with ESCs, is in charge of preserving the pluripotency and ability to self-renew. NANOG has high expression in a variety of malignancies and is involved in the carcinogenesis process, resulting in resistance to radiation and chemotherapy [5,6]. Studies have shown that prostate cancer cells acquire stem-like properties through the expression of NANOG, particularly in stable and accumulated conditions. Phosphorylation of NANOG is essential to maintain NANOG stability, thereby enhancing tumorigenic properties [7]. Polo-like kinase 1 (PLK1) functions as a serine/threonine kinase and plays a critical role as a key regulator in cell cycle progression. Notably, PLK1 has been widely recognized as an oncogene, with its overexpression associated with genomic instability. This heightened expression promotes cellular transformation and has been consistently linked to poor prognosis in cancer patients [8]. In this review, we aim to explore the potential role of PLK1 in promoting stemness and driving lineage plasticity, with a particular focus on its regulation of NANOG.

## 2. Appearance of Lineage Plasticity as A Resistance Mechanism

Multiple resistance mechanisms to AR-targeted therapies have been identified, including restoration of AR signaling, mutations in the ligand binding domain of AR and alternative splicing of AR mRNA, bypassing of AR signaling, and the independency to AR through lineage plasticity [4]. Through the development of lineage plasticity, prostate cancer cells evade AR pathway inhibitors such as enzalutamide by shedding their dependence on the AR pathway by transitioning to a stem cell-like state and acquisition of undifferentiated features and stemness properties followed by redifferentiation to new lineages such as neuroendocrine (NE)-like which is often associated with more aggressive stages of cancer [9,10]. The role of therapy-induced lineage plasticity has been highlighted previously, showing that AR-positive prostate cancer (ARPC) shifts into a stem-like phenotype in response to AR signaling inhibitors (ARSIs), such as enzalutamide, through the inactivation of TP53 [11]. Although the exact molecular mechanism that promotes lineage plasticity is not fully understood, histological analysis showed reduced AR levels and increased expression of neuroendocrine markers after relapsing from androgen-deprivation therapies (ADTs). RB1 mutation is more common in ADT-recurrent prostate cancer than primary tumors. This suggests that there is selective pressure for RB1 loss during tumor evolution [12]. When combined with the loss of TP53, this drives a transition from AR-dependent luminal epithelial cells to AR-independent basal-like cells. This transition is associated with reduced AR levels and increased expression of epigenetic reprogramming factors such as Ezh2 and SOX2, as well as the pluripotency transcription factor NANOG and neuroendocrine marker SYP. These findings underscore the critical role of TP53 and RB1 deficiencies in enabling lineage plasticity and resistance to AR-targeted therapies [12–14].

Building on these genetic alterations, evidence indicates the activation of kinase pathways to rewire transcriptional programs, leading to bypassing AR-targeted therapies and promoting cellular plasticity. Receptor Tyrosine Kinase-Like Orphan Receptor 2 (ROR2) has been identified as a regulator of lineage plasticity. Under the pressure of ENZA, ROR2 expression is upregulated, promoting a stem cell-like phenotype. ROR2 is essential for activating ASCL1, a critical transcription factor for neuroendocrine differentiation. In this case, silencing ASCL1 may reverse lineage plasticity [15].

Stemness factors such as Lin28B further contribute to lineage plasticity by repressing let-7 microRNA, fostering a stem cell-like phenotype and enabling transdifferentiation into neuroendocrine lineages in response to AR pathway inhibitors [16]. In the context of TP53 and RB1 deficiencies, activation of the JAK-STAT signaling pathway enhances stem-like properties and facilitates the transition to neuroendocrine phenotypes through the upregulation of SOX2 [17].

Additional transcriptional regulators, such as FOXA1 and FOXA2, play pivotal roles in driving neuroendocrine progression. FOXA1 has a negative correlation with NEPC progression, as its loss promotes neuroendocrine differentiation, marked by increased expression of neuroendocrine markers like ENO2. FOXA1 suppresses IL-8 transcriptional activity through direct binding to the IL-8 promoter, preventing activation of the MAPK/ERK pathway, which is crucial for neuroendocrine differentiation [18]. In contrast, FOXA2 promotes NEPC development, with single-cell multi-omics revealing that FOXA2 activates the KIT pathway and drives the adeno-to-neuroendocrine transition [19].

The anti-apoptotic protein BCL2 also plays a role in supporting neuroendocrine features and therapy resistance. BCL2 is significantly upregulated in small-cell neuroendocrine prostate cancer (SCNPC) compared to AR-positive prostate cancers (ARPCs), with its expression strongly associated with neuroendocrine characteristics and inversely related to AR activity, suggesting that BCL2 facilitates the transdifferentiation process from ARPC to SCNPC and contributes to therapy resistance [20].

Together, these findings demonstrate that ARSIs, along with genetic alterations such as the loss of RB1 and TP53, and the activation of multiple signaling pathways and transcriptional programs, contribute to the development of lineage plasticity. This process enables prostate cancer cells to transition from AR-dependent states to AR-independent and neuroendocrine-like phenotypes, highlighting the multifaceted mechanisms underlying resistance to AR-targeted therapies in advanced disease (Figure 5).

## 3. Role of CSCs in Lineage Plasticity

Cancer progression is marked by the gradual loss of differentiated phenotypes and the acquisition of stem cell-like traits. Cancer stem cells (CSCs) represent a small subpopulation within tumors with tumor-initiating properties, self-renewal, and differentiation ability, regulated by transcription factors such as NANOG, SOX2, and OCT4 [21]. The differentiation process is initiated from CSC with a phenotype similar to that of normal stem cells. Well-differentiated tumors resemble normal cells and grow slowly, while poorly differentiated ones appear abnormal, grow faster, and are more aggressive. As tumors progress, they lose their normal structure, becoming more immature and less differentiated. The transcription factors in less differentiated tumors closely resemble those found in human embryonic stem cells [22]. Ben-Porath *et al*., by analyzing the activity of gene sets linked to human ES cell identity in human tumors, demonstrated that the presence of an ES-like gene set enrichment signature in tumors is inversely related to the level of tumor differentiation. They explored the expression of ES cell-like gene signatures in poorly differentiated human tumors and found that genes associated with ES cell identity, such as NANOG, OCT4, SOX2, and c-MYC, are overexpressed in aggressive, poorly differentiated tumors, particularly in breast, glioblastoma, and bladder cancers. These ES-like signatures are linked to poor clinical outcomes and high-grade, estrogen receptor-negative breast cancers. The research highlights a correlation between stem cell-like traits in tumors and poor differentiation, suggesting that these tumors may adopt molecular features of stem cells, contributing to their aggressive behavior [23]. In concordance, Civenni *et al*. investigate the role of MYC in maintaining CSCs in prostate cancer and explore the therapeutic potential of silencing MYC transcription using RNAi. They found that silencing MYC significantly reduced the CSC population, impaired their self-renewal ability, and induced senescence. Additionally, MYC silencing suppressed tumor formation and metastasis in xenograft models, demonstrating that targeting MYC can effectively deplete the CSC compartment and inhibit tumor progression [24].

Normal and neoplastic non-stem cells can convert into stem-like cells. A subpopulation of basal-like human mammary epithelial cells has been identified as capable of spontaneous dedifferentiation into stem-like cells. Oncogenic transformation further enhances this conversion, allowing non-stem cancer cells to generate CSC-like cells both *in vitro* and *in vivo* [25]. Similarly, another study highlights that non-cancer stem cells can acquire cancer stem cell-like properties through stochastic state transitions. By examining breast cancer cell lines, the researchers show that distinct cell populations—such as basal, luminal, and stem-like states—can transition between one another, maintaining a dynamic balance of cell states. This indicates phenotypic and functional plasticity, allowing differentiated cancer cells to revert to a more stem-like state through dedifferentiation [26].

Prolonged androgen-deprived conditions have been shown to induce stemness properties in CRPC cells. ADT increases serum interferon levels, leading to the activation of the JAK-STAT pathway. STAT1-driven interferon signaling plays a significant role in promoting lineage plasticity and neuroendocrine differentiation in prostate cancer. Furthermore, IFN-induced STAT1 activation enhances prostate cancer stem cell properties [14]. In prostate cancer stem cells, AR and PSA are low or negative, and they are less differentiated. loss of AR expression promotes CSC generation, enabling prostate cancer cells to use stem cell signaling for better survival [27]. Differentiated cancer cells can gain CSC-like properties during prostate cancer progression and express little differentiation marker PSA under particular conditions [28,29]. Liu *et al*. demonstrated that poorly differentiated prostate cancer cell populations that phenotypically lack PSA expression express more stemness-related genes [21]. PSA is the most reliable marker for lineage differentiation, particularly in identifying differentiated luminal cells of the human prostate. The PSA^−^/lo prostate cancer cell population exhibits significantly higher tumorigenicity in androgen-deprived hosts than the PSA^+^ population and plays a key role in the regeneration of CRPC [30].

A negative correlation between AR and stemness has been reported. In stem-like AR^−^ CSCs, MDM2 promotes the constant degradation of AR protein, thereby maintaining prostate CSC pluripotency and inhibiting epithelial cell lineage specification. AR is not required for prostate CSC or normal prostate progenitor/stem cell function. Inhibition of MDM2 leads to AR expression, causing CSC differentiation into luminal epithelial cells [31]. Furthermore, depletion of AR signaling has been linked to prostate cancer cell reprogramming, driving them toward the cancer stem-like stage, as evidenced by increased levels of NANOG and OCT4. Building on this AR-independent state, the transcription factor SIX2 plays a key role in AR-independent prostate cancer, promoting cancer cell plasticity, stemness, and CSC-like properties through Wnt/β-catenin signaling, particularly following AR inhibition by enzalutamide. SIX2 expression is elevated in AR-negative and neuroendocrine prostate cancers, while SIX2 depletion reduces cancer cell proliferation, invasion, migration, and stemness [32].

## 4. NANOG as a Master Regulator of Stemness

Cancer stem-like cells are characterized by the expression of pluripotency-associated genes like NANOG, OCT3/4, and SOX2, which serve as key transcription factors required to preserve the characteristics of ES cells [33]. Immunohistochemical expression of stem cell markers, including NANOG, in prostate cancer biopsies from 114 patients showed that NANOG expression was significantly higher in prostate adenocarcinoma cells compared to non-cancerous prostate cells and increased with higher Gleason scores. The findings suggest a correlation between NANOG expression and cancer aggressiveness, as indicated by the Gleason score [34]. The NANOG gene family includes 11 known paralogs, with NANOG1 being prominently expressed in human embryonic stem cells, where it plays a pivotal role in maintaining pluripotency. Among these paralogs, NANOGP8 stands out as the only intronless variant capable of producing a functional protein, differing from NANOG1 by just two amino acids located within domains critical for transcriptional regulation. These differences suggest potential functional distinctions between NANOGP8 and NANOG1. Remarkably, NANOGP8 is widely expressed across various human cancer cell lines, contributing to properties associated with cancer stem cells, such as clonogenicity and tumorigenicity. Quantitative analyses demonstrate that while NANOG1 expression is minimal in cancer cells, NANOGP8-derived protein levels are comparable to NANOG1 in pluripotent cells. Functional studies reveal that NANOGP8, like NANOG1, enhances reprogramming efficiency in both murine and human fibroblasts [35,36]. The N-terminus is rich in serine, threonine, and proline, forming a structural motif that supports NANOG’s transcriptional activity, which is tightly regulated by phosphorylation or other post-translational modifications [37]. The predicted molecular weight of NANOG is 35 kD; however, studies have shown that NANOG protein migrates at multiple molecular weights (ranging from 29 kD to 80 kD) on SDS-PAGE, with a predominant band at 42 kD in NTERA-2 cells based on siRNA-mediated knockdown. Eight different anti-NANOG antibodies exhibited differential reactivity toward these proteins, indicating that NANOG can exist in multiple conformations [35].

NANOG transcription is regulated by the combined action of OCT4 and SOX2, which bind to a specific regulatory region in its promoter known as the SOX-OCT element. This region, located upstream of the NANOG gene, is essential for its expression in pluripotent cells. The interaction between OCT4 and SOX2 forms a complex that activates NANOG, with studies showing that reducing either factor significantly lowers NANOG expression. This regulatory sequence is conserved across mammals, emphasizing its critical role in maintaining the precise expression needed for pluripotency. However, additional factors may also contribute to NANOG activation, suggesting a more complex regulatory mechanism [38].

Previous studies have confirmed a negative correlation between p53 and NANOG. P53 can directly bind to the NANOG promoter and negatively regulate its expression. The loss of p53 promotes reprogramming and lineage plasticity by increasing NANOG levels. Notably, Gli-mediated regulation of NANOG, driven by the Hedgehog (Hh) signaling pathway, operates independently of p53. Activation of Hh signaling triggers the release of Smoothened (Smo) inhibition by Patched1 (Ptc1), enabling downstream effectors Gli1 and Gli2 to modulate NANOG expression. Gli2 is the initial activator, binding specific response elements in the NANOG promoter to initiate transcription. Subsequently, Gli1, induced as a downstream target of Hh signaling, replaces Gli2 to sustain and enhance NANOG transcription. This dynamic interaction is accompanied by histone H3 acetylation at the promoter, facilitating an open chromatin state for active transcription. Notably, Hh signaling can override p53-mediated repression of NANOG. These mechanisms highlight the essential role of Gli proteins in integrating signaling cues to control NANOG expression [39,40].

Regulation of NANOG transcription involves multiple pathways that converge on its promoter and enhancer regions. In mouse embryonic stem cells, E-Cadherin facilitates a cadherin-dependent pathway wherein its β-catenin-binding domain enables the phosphorylation of STAT3. Phosphorylated STAT3 then directly interacts with the NANOG promoter to enhance transcription. Simultaneously, the Wnt/β-catenin signaling pathway plays a critical role by allowing β-catenin, in conjunction with Lef1, to bind specific Tcf/Lef sites in the NANOG enhancer region, particularly between −1020 and −1004. This activation is further amplified by inhibiting GSK3β, which prevents β-catenin degradation, and by proteins like Dishevelled-1 (Dvl-1), which reinforce β-catenin activity. Together, these pathways highlight the integration of cadherin- and Wnt-mediated signaling mechanisms in the precise control of NANOG expression [41,42].

Distinct promoter regions, termed CR2 and CR1, play specialized roles in the transcriptional regulation of NANOG. CR2 serves as a binding site for ESE-1, an epithelium-specific ETS transcription factor. ESE-1 acts as a transcriptional repressor by binding to CR2, suppressing NANOG expression and inhibiting promoter activity. Conversely, ESE-1 knockdown enhances NANOG transcription. This repressive effect is dependent on ESE-1’s transactivation domain, as its deletion abrogates repression. Additionally, SMAD2/3, activated by TGFβ/Activin signaling, directly regulates NANOG transcription via CR1 promoter sites. Phosphorylated SMAD2/3 binding to CR1 enhances NANOG transcription, while mutations in these binding sites significantly impair promoter activity. Together, these findings emphasize the distinct yet complementary regulatory roles of CR2 and CR1 in controlling NANOG expression, which is critical for cellular pluripotency and tumorigenesis [43,44] (Figure 1).

A potential mechanism for NANOG regulation involves post-translational modifications, with phosphorylation being particularly significant. Moretto-Zita *et al*. reported that NANOG can be phosphorylated at Ser52 and Pro65, which are critical for the interaction of Pin1 to NANOG. This interaction is essential for NANOG stabilization by inhibiting its degradation in a proteasome-dependent manner, as the binding of Pin1 to NANOG inhibits its ubiquitination. Disruption of the binding between NANOG and Pin1 inhibits the self-renewal of ESCs. Although the specific kinase responsible for this phosphorylation has yet to be identified, it has been proposed that cyclin-dependent kinases and mitogen-activated protein kinases are the primary kinases responsible for NANOG phosphorylation, implying that the cell cycle may regulate NANOG protein levels [45].

In another study, mass spectrometry analysis showed that ERK2, an important downstream mediator in the FGF signaling pathway, directly phosphorylates NANOG, leading to increased NANOG stability [46]. Additionally, BRAF phosphorylates NANOG at Ser68. The wild-type form of BRAF, but not the kinase-dead mutant K483M, significantly increased the protein level and prolonged the half-life of NANOG, promoting prostate cancer stem cell-like traits. Conversely, BRAF knockdown decreased NANOG protein levels through the interaction between SPOP and NANOG, which promotes NANOG ubiquitination [47]. Given the upregulation of BRAF under androgen-independent conditions, this phosphorylation likely contributes to CRPC progression [48].

Further research demonstrates that phosphorylation of NANOG at residues T200 and T280, mediated by PKCε, is crucial for its stability and function in tumorigenesis, particularly within head and neck squamous cell carcinoma. Mutant NANOG proteins lacking these phosphorylation sites exhibited impaired homodimerization, reduced DNA binding, and a dominant-negative effect on endogenous NANOG activity. This resulted in decreased cell proliferation, invasion, and cancer-initiating cell populations. Furthermore, NANOG directly regulates Bmi1 by binding to its promoter, and this NANOG-Bmi1 axis is critical for promoting tumorigenesis [49] (Figure 2).

One key difference between differentiated cells and stem cells is the significantly shorter G1 phase in the cell cycle of stem cells [50]. Coronado *et al*. reported that a shortened G1 phase supports pluripotency. They demonstrated this by showing that cyclin E overexpression, which accelerates the G1-S transition, enhances stemness. In contrast, cyclin E knockdown prolongs the G1 phase, promoting differentiation [51]. In another study, van der Laan *et al*. demonstrated the role of NANOG in cell cycle-associated events. NANOG expression in mouse ESCs displays strong fluctuations that depend on the cell cycle, with changes in NANOG levels occurring during different phases [52]. Zhang *et al*. found that NANOG drives the transition from the G1 to the S phase. NANOG accomplishes this by transcriptionally activating two critical cell cycle regulators: CDK6 and CDC25A [50] (Figure 3).

Overexpression of NANOG accelerates entry into the S phase, while its downregulation delays this process. CDK6 and CDC25A are essential downstream effectors of NANOG, as their knockdown prevents the NANOG-driven acceleration of S-phase entry. When NANOG is downregulated, it causes delays in the S-phase and the transition from G2 to M. CDC25A plays a role in these transitions, and other regulatory components likely mediate NANOG’s effects on S-phase and G2 to M progression in human ESCs [50]. In another study, Gonzales *et al*. examined the role of the cell cycle in regulating stemness and cell fate in pluripotent cells. They found that NANOG is preferentially transcribed during the S and G2-M phases of the cell cycle, highlighting the importance of these phases in maintaining pluripotency tendency. When the cell cycle is arrested in these phases, it leads to a delay in the shutdown of pluripotency and an increase in NANOG levels, resulting in delayed differentiation [53].

Several studies have addressed the role of NANOG in prostate cancer progression. Jeter *et al*. demonstrated that NANOG is upregulated following castration and is essential for sustaining CRPC. NANOG suppresses AR-regulated pro-differentiation genes, contributing to resistance. It binds to the AR/FOXA1 genomic regions and interacts directly with AR and FOXA1, disrupting the transcription of AR-controlled genes, which results in a loss of differentiation while simultaneously inducing stem cell-like characteristics. Additionally, NANOG activates MYC, driving increased cell proliferation and further promoting the transition to CRPC [6].

A different study emphasized the role of NANOG gain of function in contributing to resistance against androgen deprivation. The researchers observed that short-term overexpression of NANOG did not affect cell proliferation; however, prolonged NANOG induction under androgen-deprived conditions led to increased sphere formation in LNCaP cells. NANOG expression resulted in significantly larger tumors in castrated mice compared to non-castrated controls. Additionally, NANOG induction in LNCaP androgen-independent tumors in castrated mice exhibited increased proliferation, as evidenced by Ki-67 staining, compared to non-castrated mice. The study also identified an inverse relationship between NANOG and AR/PSA levels under androgen deprivation. One proposed mechanism for NANOG-mediated castration resistance involves the upregulation of the anti-apoptotic protein Bcl-2, whose elevated expression is strongly correlated with the progression of CRPC [5]. In another study, loss-of-function experiments revealed that human prostate cancer cells infected with a control vector recombined with rUGSM cells and transplanted under the renal capsule of NOD/SCID mice exhibited significant outgrowth compared with those infected with NANOG-shRNA, which did not regenerate any outgrowth. This indicates that the loss of NANOG significantly impairs the tumorigenic potential of human prostate cancer cells, supporting the role of NANOG in promoting tumor growth [54].

Another study highlights the critical role of stemness features in driving the lethality of AR/enhancer-altered mCRPC. Genome-wide methylation sequencing of plasma cell-free DNA revealed that transcription factor binding sites associated with stemness were significantly more accessible in lethal AR-altered mCRPC cases. This increased accessibility indicates an epigenomic reprogramming that favors stem-like properties closely linked to cancer aggressiveness and therapeutic resistance. A key finding was identifying a 20-gene stemness signature in plasma cfDNA that predicted worse survival outcomes, suggesting that these stem-like features are a primary mechanism behind the lethality of AR/enhancer-altered prostate cancers [55].

Kainulainen *et al*. explored the role of M1 macrophages in driving cancer stem cell plasticity and demonstrated that AR inhibition is closely associated with increased NANOG expression. Their protein network analysis revealed that pro-inflammatory cytokines, particularly IL-6 and TNFα, secreted by M1 macrophages, influence the expression of NANOG and AR/PSA. In prostate cancer, these secreted factors stimulate CSC markers like NANOG while suppressing AR signaling. This leads to the reprogramming of prostate cancer cells into a more stem-like phenotype, as evidenced by higher levels of NANOG and diminished AR activity [56].

AR+ LNCaP cells grow better in the presence of androgen than AR-KO cells. AR+ clones also generate larger tumors in androgen-abundant environments. However, in androgen-ablated hosts, such as castrated mice, AR-KO cells surpass AR+ cells in tumorigenic capacity, forming larger and more frequent tumors. These findings suggest that AR+ prostate cancer cells grow well in the presence of androgens. In contrast, AR-KO cells possess an inherent advantage under androgen-deprived or ENZA-treated conditions, showcasing their resistance to anti-androgen therapies. Moreover, Bcl-2 contributes to ENZA resistance, particularly in AR+/hi CRPC cells, which enhances the tumorigenic and stem cell-like properties of prostate cancer cells, supporting their survival and proliferation under anti-androgen therapy. Additionally, gene expression analysis reveals enrichment of stem cell signaling pathways in AR+/hi CRPC, linking stem cell-like traits to therapeutic resistance. AR-KO LNCaP cells show a higher rate of cell-cycle progression in ENZA than DHT, indicating that androgens may inhibit AR-KO cells [57]. Thus, the loss of AR expression promotes a stem-like cell phenotype, implying an inverse relationship between AR signaling and prostate cancer stemness. There is increased accessibility to transcription factors associated with stemness in lethal AR-altered mCRPC cases, suggesting that as AR signaling is disrupted or altered, there is a shift toward a more stem-like, undifferentiated state. This supports the notion that diminished AR signaling may drive or enhance stem-like characteristics in prostate cancer, which are linked to aggressiveness, therapeutic resistance, and poor prognosis.

Building on these findings, innovative therapeutic approaches have been explored to target NANOG effectively, supported by studies highlighting its critical role in regulating stemness in glioblastoma. Since NANOG is generally absent in differentiated somatic cells, therapies targeting it are less likely to cause off-target effects. However, as transcription factors like NANOG are challenging to inhibit using small molecules or antibodies, alternative strategies, such as chimeric repressors, have been developed. One such approach is the creation of chimeric NANOG repressor proteins, termed NANEPs, designed to block endogenous NANOG function. NANEP5, the most effective variant, mimics the effects of NANOG knockdown both *in vitro* and *in vivo*, driving profound transcriptional and cellular remodeling that disrupts tumor growth and persistence. This strategy shows potential for further development, particularly in combination with existing treatments [58].

## 5. PLK1 Regulation of Stemness and Cell Plasticity

PLK1, a well-known serine/threonine kinase and a member of the Polo-like kinase (PLK) family, has been demonstrated to play crucial roles in mitosis and cytokinesis. PLK1 facilitates mitotic cell division and is essential in driving accelerated cell proliferation. PLK1 overexpression allows tumor cells to bypass mitotic checkpoints, leading to genetic instability and the transformation of mammalian cells. A comparison of PLK1 expression between cancerous and normal tissues revealed significantly higher PLK1 expression in cancer tissues compared to normal tissues [59]. The PLK1 protein consists of two polo-box domains (PBD) and a kinase domain. The PBD serves as a docking site that depends on phosphorylation to bring the kinase closer to its target. It is also crucial for the precise subcellular positioning of PLK1, as its substrates must be positioned near the PBD for effective interaction. As PLK1 accumulates at the mitotic centrosomes, kinetochores, and the cytokinetic midbody in a spatially and temporally regulated manner, its substrates are anticipated to colocalize at these sites. When PLK1 substrates undergo an initial phosphorylation, either by CDK1 or PLK1 itself, it enhances the interaction between PLK1 and its substrates [60,61]. Once activated, PLK1 expression increases during the S phase, peaks at the G2-M transition, and rapidly decreases as the cell exits mitosis [62].

Previous studies have confirmed that PLK1 positively regulates AR signaling, and its elevation leads to the constitutive activation of AR signaling, contributing to ENZA resistance [63]. The role of PLK1 as a tumor promoter has been emphasized in several studies. PLK1 promotes p53 degradation through multiple mechanisms, and cells that have lost p53 and exhibit elevated PLK1 expression levels are at an increased risk of undergoing oncogenic transformation [64]. Additionally, PLK1 regulates the stability of various oncogenes. Mo *et al*. demonstrated that MYC oncoprotein stabilization is PLK1-dependent. Inhibition of PLK1 using BI2536 reduces MYC stabilization, thereby limiting MYC-driven cell proliferation [65]. It has also been reported that overexpression of MYC promotes androgen-independent prostate cancer progression [66].

Mai *et al*. investigated the role of PLK1 phosphorylation in stemness and the promotion of advanced nasopharyngeal carcinoma (NPC). They found that PLK1 directly phosphorylated KLF4 at Ser234, leading to the stabilization and upregulation of KLF4 by promoting the recruitment and attachment of the E3 ligase TRAF6 to KLF4. This interaction results in K63-linked ubiquitination and stabilization of KLF4. The enhanced stability of KLF4 increases its oncogenic potential in NPC [67]. Previous studies have highlighted the role of FOXM1 in the G2/M transition and proper mitotic progression through its interaction with β-catenin. Additionally, the interaction between FOXM1 and β-catenin enhances the self-renewal and tumorigenic potential of glioblastoma-initiating cells [68]. FOXM1 has also been implicated in maintaining the pluripotency of stem cells, with a positive correlation observed with stemness genes such as NANOG [69]. Fu *et al*. reported that CDK1 first phosphorylates FOXM1, creating a docking site for the PLK1 PBD domain. This allows PLK1 to bind to FOXM1 and phosphorylate it at S715 and S724 during late G2/M, leading to the subsequent activation of FOXM1 activity [70] (Figure 4).

Dimri *et al*. demonstrated that BMI1, a crucial regulator of CSCs, can be regulated by PLK1 and that inhibiting PLK1 suppresses the CSC phenotype in breast cancer cells. They suggest using PLK1 inhibitors to eliminate the CSC population and overcome therapy resistance [71]. Another study explores the connection between the oncoprotein CIP2A and AR in prostate cancer. The findings reveal that CIP2A is highly expressed in prostate cancer tissues and is associated with increased AR levels, which contribute to cancer progression. AR signaling regulates CIP2A expression, and in return, CIP2A modulates AR levels, enhancing cell proliferation and resistance to treatments such as ENZA. The study also identifies that PLK1 activity is necessary for CIP2A regulation of AR, suggesting that targeting CIP2A could be a promising therapeutic strategy for CRPC [72].

Another study identified FOXC2 as a regulator of stem cell traits, particularly in controlling the cell cycle of CSC-enriched breast cancer cells. The research revealed that FOXC2 expression follows a cell cycle-dependent pattern, with protein levels rising during the G2 phase and rapidly dropping during mitosis. The stability of FOXC2 is heavily dependent on PLK1 activity, as inhibiting PLK1 leads to a reduction in FOXC2 levels. In nocodazole-treated cells, those in late G2 showed higher FOXC2 levels, whereas prometaphase-enriched cells displayed significantly lower levels. This suggests a role for FOXC2 in managing the G2/M transition. Additionally, FOXC2 acts downstream of PLK1, with PLK1 being essential for FOXC2 stability and supporting CSC properties such as self-renewal and sphere formation [73]. Li *et al*. demonstrated that PLK1 sustains stemness by regulating SOX2 expression in EGFRvIII-positive glioma stem cells. Inhibition of PLK1 or its knockdown reduced SOX2 expression, thereby diminishing the self-renewal capacity of glioma stem cells [74]. Our unpublished data demonstrate that NANOG and PLK1 physically interact, with PLK1 playing a regulatory role in enhancing NANOG stability in prostate cancer cell lines. Immunofluorescence staining reveals the co-localization of NANOG and PLK1, further supporting their interaction in a cell cycle-dependent manner. Aligned with these findings, in papillary thyroid carcinoma and triple-negative breast cancer, PLK1 knockdown or inhibition with volasertib significantly reduces spheroid growth and the expression of stem cell markers, including NANOG, CD133, and CD44. Conversely, overexpression of PLK1 enhances spheroid formation and upregulates NANOG along with other stem cell markers. These findings highlight a functional connection between PLK1 activity and NANOG expression, emphasizing PLK1 critical role in promoting the stemness phenotype and driving lineage plasticity [75,76].

## 6. Conclusions and Perspectives

The advancement and clinical efficacy of stronger AR signaling inhibitors have been accompanied by a rise in aggressive AR-negative prostate cancers. The emergence of lineage plasticity as a resistance mechanism is expected to grow further with the introduction of powerful ARSIs. The multifactorial nature of lineage plasticity in prostate cancer underscores the complexity of resistance mechanisms. This phenomenon engages a combination of tumor suppressor gene mutations, epigenetic reprogramming, transcriptional alterations, and metabolic shifts. Prostate cancer cells, under the selective pressures of AR inhibitors and androgen deprivation, reactivate developmental programs to enhance phenotypic plasticity, allowing them to evolve toward more aggressive lineages such as neuroendocrine prostate cancer. Understanding the precise mechanisms underlying lineage plasticity is crucial for addressing more aggressive forms of prostate cancer.

Loss of tumor suppressors like RB1 and p53 has been shown to facilitate lineage plasticity and the emergence of NEPC. Concurrently, overexpression of stemness factors such as SOX2 is necessary for this progression and lineage shift. Notably, increased levels of NANOG have been observed in sgTP53/RB1 LNCaP cells, further supporting this process. Kinase pathway activation is crucial in reprogramming transcriptional networks, allowing cancer cells to evade AR-targeted therapies and enhance cellular plasticity (Figure 5).

PLK1 is an essential cell cycle regulator, with its levels rising notably during the G2/M transition and M phase. It is also frequently overexpressed in cancerous tissues because of increased cell proliferation. The role of PLK1 in promoting the degradation of p53 and its increased expression in p53-deficient cells, combined with its regulation of the stability of various oncogenes and stemness-related genes, underscores PLK1’s significant contribution to driving lineage plasticity.

PLK1-mediated stabilization of NANOG underscores its involvement in CRPC progression through a phosphorylation-dependent mechanism that drives early reprogramming events. Prostate cancer cell populations with upregulated and stabilized NANOG through PLK1 kinase activity, suppresses AR-regulated pro-differentiation genes and exhibit a less differentiated phenotype, characterized by PSA^−^/low cells that are quiescent compared to PSA^+^ cells. Under the selective pressure of ADT and enzalutamide, sensitive cell populations fail to survive, while the quiescent cell population gains sufficient time to undergo reprogramming, enabling survival. These reprogrammed and differentiated cells subsequently become the dominant population and rapidly proliferate.

These events intersect with the pivotal role of AR in modulating the balance between self-renewal and differentiation. Under therapeutic pressure, cells exploit this balance to adopt a stem-like state, promoting CRPC progression. These findings, along with the regulatory interplay between FOXA1, AR, NANOG, and PLK1 highlight fundamental mechanisms facilitating lineage plasticity in transitioning from AR dependence to more aggressive AR-independent prostate cancer.

Establishing a novel system to mimic the transition from AR-positive to AR-negative states could provide critical insights into the molecular characteristics underlying this process. While AR repression facilitates cellular reprogramming and transdifferentiation to NEPC, the acquisition of neuroendocrine characteristics may represent a spectrum of differentiation from ARPC to NEPC, rather than an inevitable outcome of AR ablation. The complexity of this regulatory network, potentially involving unidentified factors, underscores the need for further investigation to fully understand and address this lethal progression. Accordingly, CRISPR-based whole-genome knockout screens could serve as a powerful tool to identify key regulators and pathways driving this transition.

## Figures and Tables

**Figure 1.**
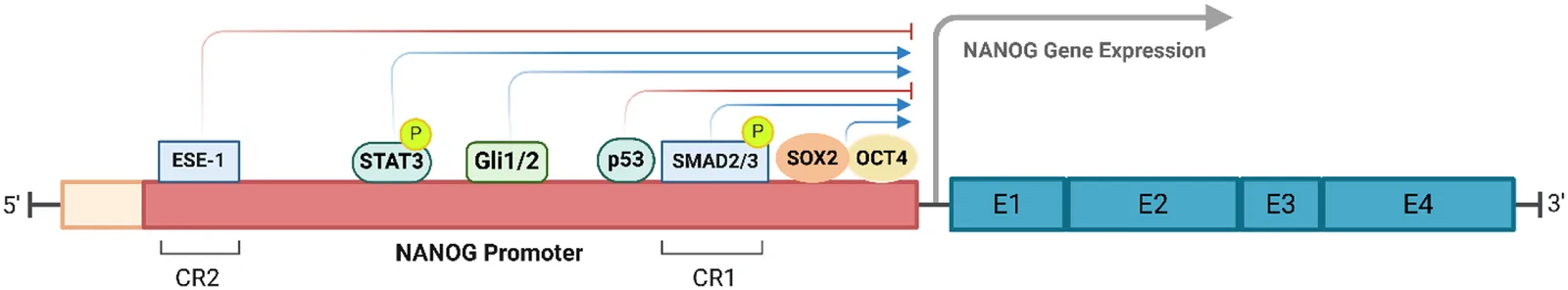
Transcriptional regulation of NANOG. Created in BioRender. Maasoumy H. (2024)

**Figure 2.**
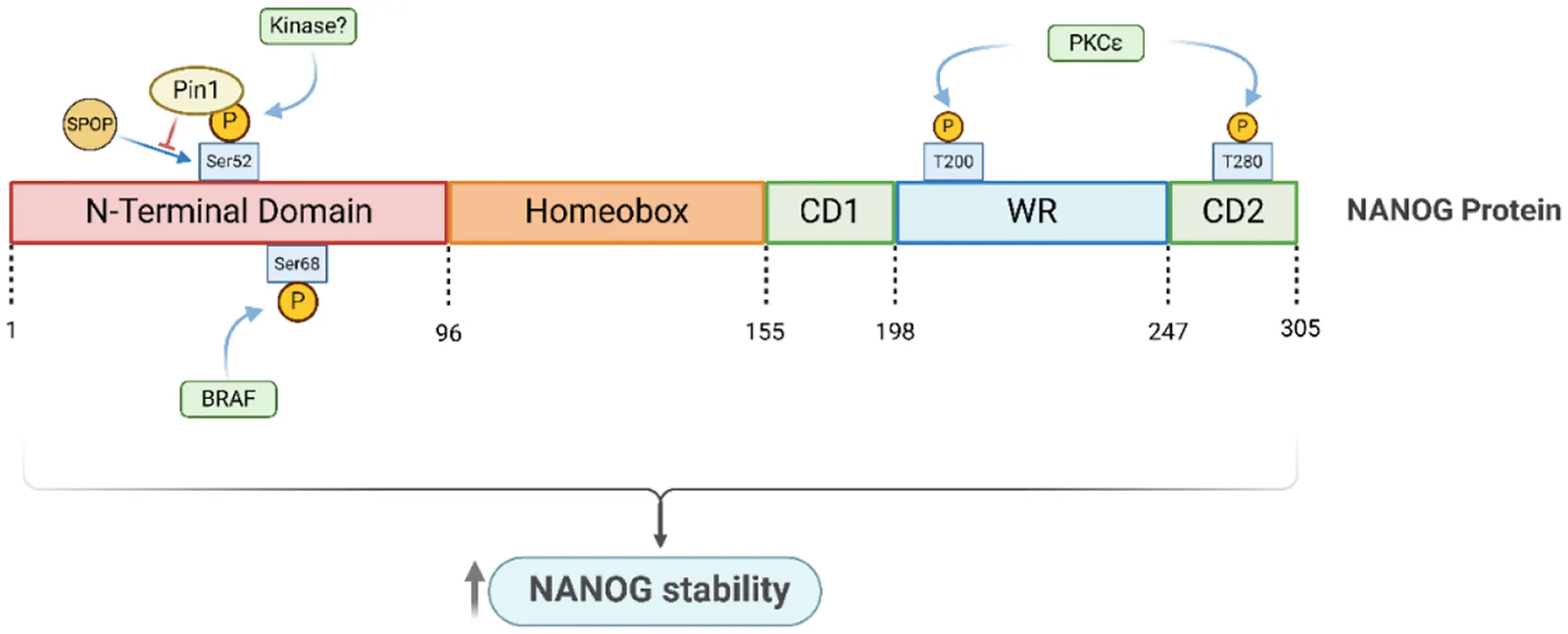
NANOG phosphorylation promotes protein stabilization. Created in BioRender. Maasoumy H. (2024)

**Figure 3.**
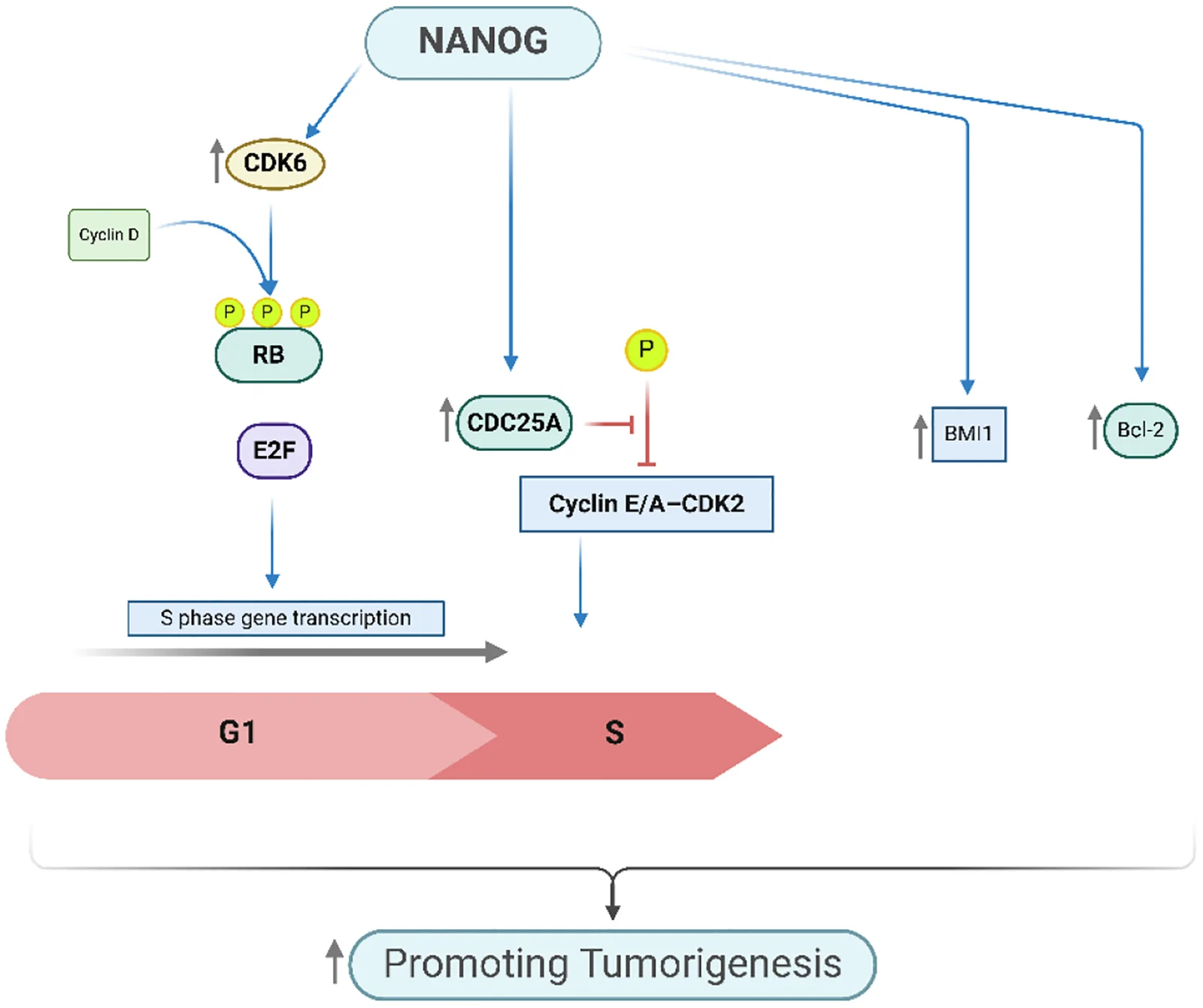
Genes modulated by NANOG contribute to tumorigenesis. Created in BioRender. Maasoumy H. (2024)

**Figure 4.**
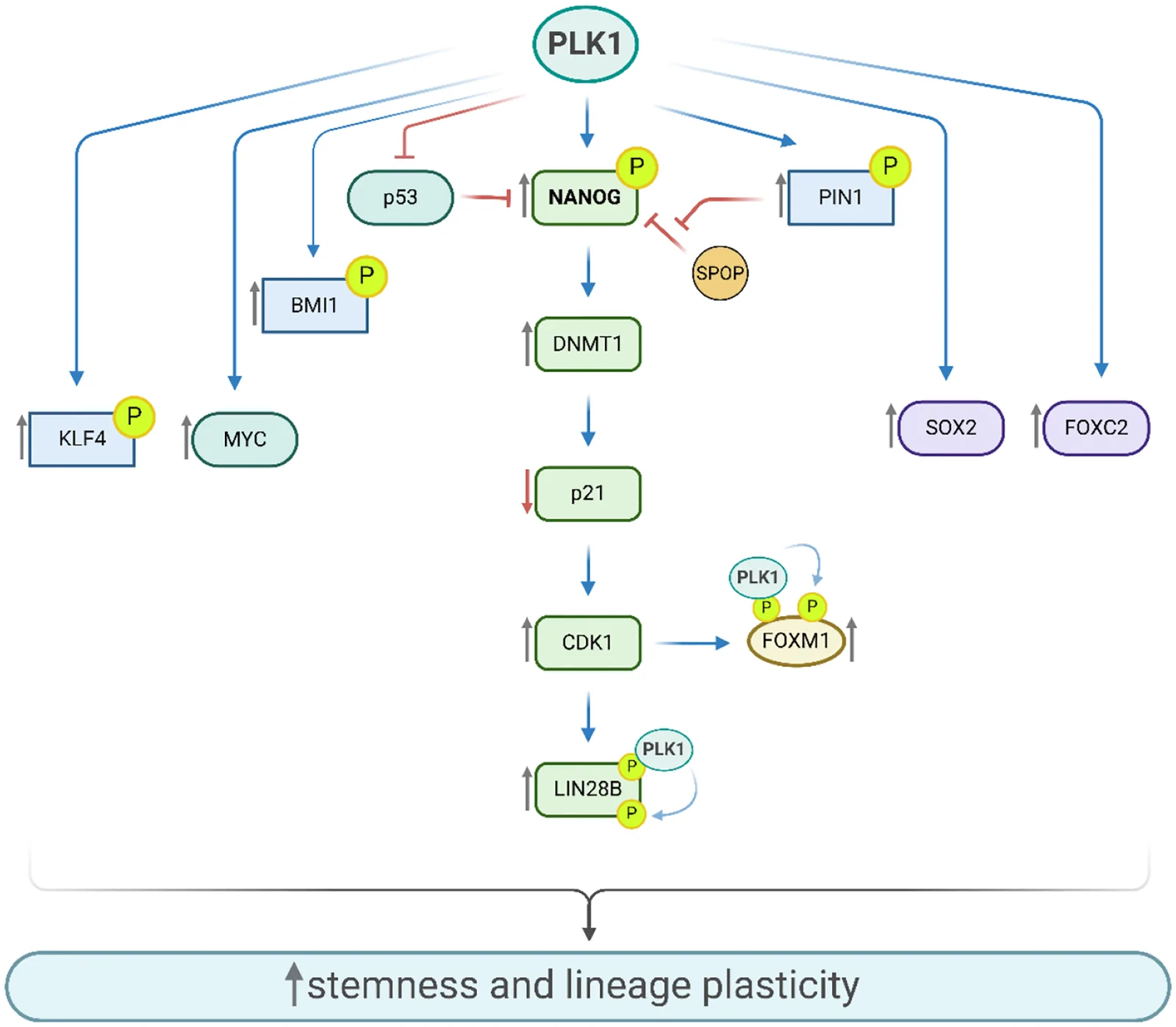
Role of PLK1 in regulating transcription factors and driving cellular plasticity in prostate cancer. Created in BioRender. Maasoumy H. (2024)

**Figure 5.**
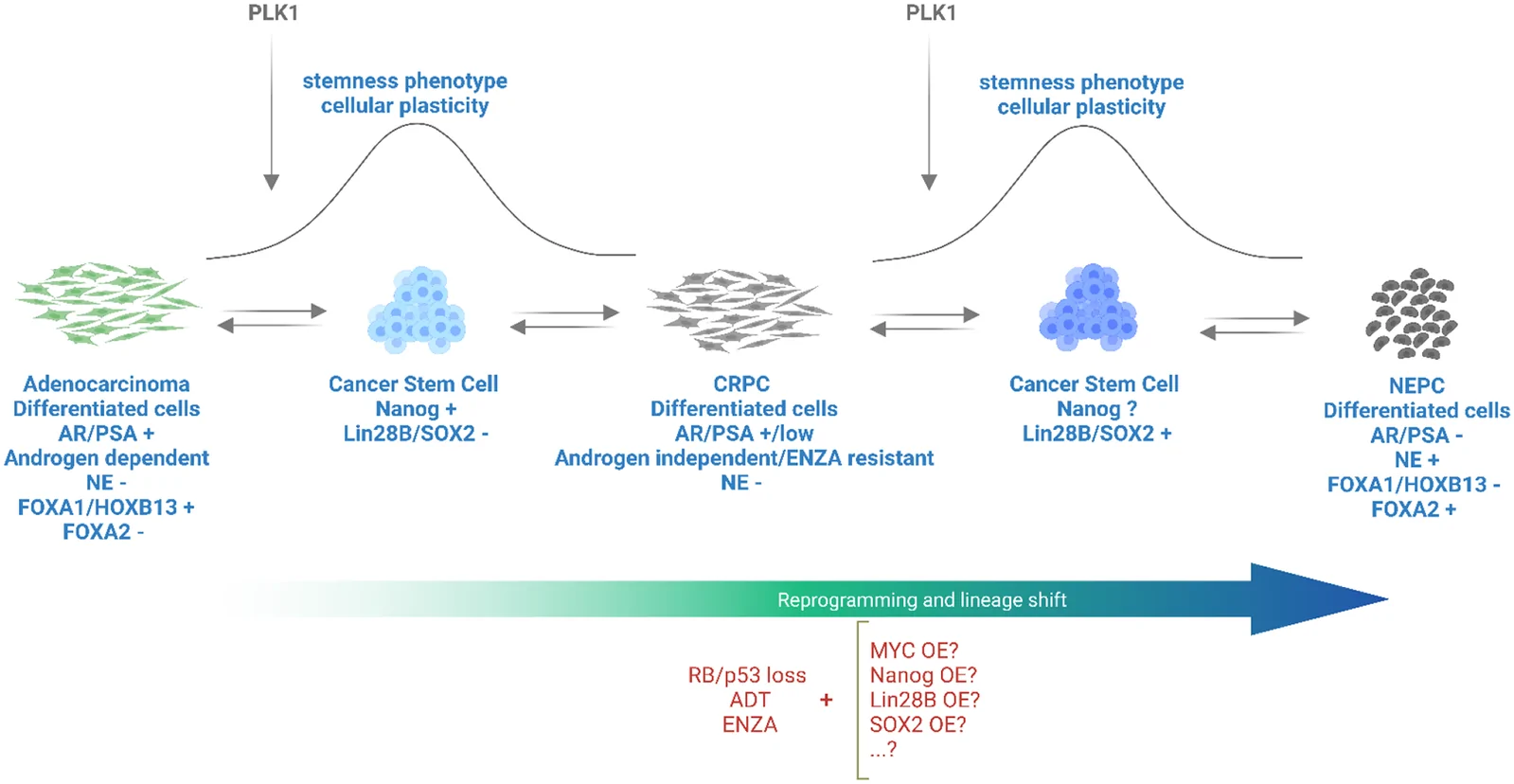
Lineage plasticity and reprogramming in prostate cancer progression. Created in BioRender. Maasoumy H. (2024).

## Abbreviations

AR: androgen receptor
CRPC: castration-resistant prostate cancer
ENZA: enzalutamide
PTM: post-translational modification
NEPC: neuroendocrine prostate cancer
CSCs: cancer stem cells
PLK1: polo-like kinase 1
ARPIs: Androgen Receptor Pathway Inhibitors
ESCs: Embryonic stem cells
NE: neuroendocrine
ARPC: AR-positive prostate cancer
ARSIs: AR signaling inhibitors
ADTs: androgen-deprivation therapies
ROR2: Receptor Tyrosine Kinase-Like Orphan Receptor 2
SCNPC: small-cell neuroendocrine prostate cancer
Hh: Hedgehog
Smo: Smoothened
Ptc1: Patched1
Dvl-1: Dishevelled-1
ESE: epithelium-specific ETS
UGSM: Urogenital sinus mesenchymal
cfDNA: cell-free DNA
AR-KO: Androgen Receptor Knockout
DHT: Dihydrotestosterone
mCRPC: metastatic Castration-Resistant Prostate Cancer
PBD: polo-box domain
AI: androgen-independent
AD: androgen-dependent
NPC: nasopharyngeal carcinoma
EGFR: Epidermal Growth Factor Receptor
